# Improved Carotid Elasticity but Altered Central Hemodynamics and Carotid Structure in Young Athletes

**DOI:** 10.3389/fspor.2021.633873

**Published:** 2021-03-15

**Authors:** Lisa Baumgartner, Heidi Weberruß, Katharina Appel, Tobias Engl, Daniel Goeder, Renate Oberhoffer-Fritz, Thorsten Schulz

**Affiliations:** Institute of Preventive Pediatrics, TUM Department of Sport and Health Science, Technical University of Munich, Munich, Germany

**Keywords:** arterial stiffness, arterial elasticity, carotid intima-media thickness, young athletes, exercise

## Abstract

Young athletes most often exceed the physical activity recommendations of the World Health Organization. Therefore, they are of special interest for investigating cardiovascular adaptions to exercise. This study aimed to examine the arterial structure and function of young athletes 12–17 years old and compare these parameters to reference values of healthy cohorts. Carotid intima-media thickness (cIMT), carotid diameter, cIMT÷carotid diameter-ratio (cIDR), arterial compliance (AC), elastic modulus (Ep), β stiffness index (β), and carotid pulse wave velocity (PWVβ) were determined using ultrasound in 331 young athletes (77 girls; mean age, 14.6 ± 1.30 years). Central systolic blood pressure (cSBP) and aortic PWV (aPWV) were measured using the oscillometric device Mobil-O-Graph. Standard deviation scores (SDS) of all parameters were calculated according to German reference values. The 75th and 90th percentiles were defined as the threshold for elevated cIMT and arterial stiffness, respectively. Activity behavior was assessed with the MoMo physical activity questionnaire, and maximum power output with a standard cardiopulmonary exercise test. One-sample *t*-tests were performed to investigate the significant deviations in SDS values compared to the value “0”. All subjects participated in competitive sports for at least 6 h per week (565.6 ± 206.0 min/week). Of the 331 young athletes, 135 (40.2%) had cIMT >75th percentile, 71 (21.5%) had cSBP >90th percentile, and 94 (28.4%) had aPWV>90th percentile. We observed higher cIMT SDS (*p* < 0.001), cIDR SDS (*p* = 0.009), and AC SDS (*p* < 0.001) but lower β SDS (*p* < 0.001), Ep SDS (*p* < 0.001), and PWVβ SDS (*p* < 0.001) compared to the reference cohort. The cSBP SDS (*p* < 0.001) and aPWV SDS (*p* < 0.001) were elevated. In conclusion, cIMT and cIDR were higher in young athletes than in a reference cohort. Furthermore, young athletes presented better carotid elasticity and lower arterial stiffness of the carotid artery. However, central arterial stiffness was higher compared to the reference cohort. The thickening of the carotid intima-media complex in combination with a reduction in arterial stiffness indicates a physiological adaptation to exercise in youth.

## Introduction

Regular physical activity reduces the risk of mortality and the incidence of cardiovascular disease worldwide (Lear and Yusuf, 2017). In youth, being active is associated with normal blood pressure (BP) and normal weight status (Leary et al., 2008). On the other hand, inactivity in youth is associated with elevated BP, which predicts hypertension in adulthood (Chen and Wang, 2008; Leary et al., 2008). Furthermore, inactivity increases the risk of being overweight or obese, which is also associated with hypertension (Chen and Wang, 2008; Juhola et al., 2011). Overweight or obese children and adolescents are more likely to be overweight or obese as adults (Singh et al., 2008; Juhola et al., 2011). The promotion of a healthy lifestyle is important in avoiding adverse effects and strengthening health resources. Therefore, the World Health Organization (WHO) recommends moderate to vigorous physical activity of at least 60 min per day in children and adolescents (World Health Organization, 2020).

The Windkessel function explains the way elastic arteries convert the pulsating blood ejections from the heart into a steady blood flow. Elastic arteries passively expand due to the BP and temporarily store some blood volume. During diastole, elastic arteries retract, thereby pushing the stored blood volume along the arterial tree. With increasing arterial stiffness, the heart has to work harder to overcome higher pressure in the vasculature. Thus, long-term increases in arterial stiffness can lead to left ventricular (LV) hypertrophy and LV failure (Luft, 2012). Arterial wall thickness and arterial compliance within the normal range are indicators of vascular health. Within the intima-media complex of the arterial wall, the tunica media is the thickest layer of arteries and consists of vascular smooth muscle cells (VSMC), collagen, and elastin (Paneni, 2017). VSMC regulate contraction of the artery and the elastic recoil of arteries in different hemodynamic situations (Basatemur et al., 2019). The tunica intima consists of the endothelium and basement membrane. The thickening of the intimal layer is a preliminary stage of atherosclerosis (Insull, 2009). This stage is characterized by the clonality of VSMC in the intimal layer, whereby VSMC seem to originate from the medial layer (Kaur et al., 2017; Basatemur et al., 2019).

Physical activity leads to a physiological process because it increases blood flow and, therefore, enhances shear stress. The increased shear stress induces prostacyclin release and endothelial nitric oxide synthase (eNOS) activity leading to increased NO production. NO inhibits the release of the vasoconstrictor, endothelin, resulting in exercise-induced vasodilation and, thus, improved vascular function. Physical activity also improves the morphometry of conduit arteries; arterial diameter increases, the dilating capacity is improved, and wall thickness is reduced (Green et al., 2017). Therefore, regular physical activity and exercise are effective primary and secondary prevention strategies to reduce arterial wall thickness and arterial stiffness, especially in at-risk populations e.g., overweight and obese patients (Baumgartner et al., 2020).

Araújo and Scharhag (2016) defined young athletes as between 12 and 17 years old, who practice exercise training to improve their performance, participate in sports competitions, are registered in sports federations, and practice their sport as a major way of living. Young athletes most often exceed the WHO physical activity recommendations and, therefore, are of special interest for investigating cardiovascular adaptions to exercise (Sharma et al., 2002). Bjerring et al. (2018) investigated 76 pre-adolescent cross-country skiers and found increased LV end-diastolic volume, LV mass, right ventricular (RV) basal diameter, and RV area. Furthermore, De Luca et al. (2011) observed higher LV diastolic diameter in 50 cyclists, soccer and basketball players with a mean age of 18.5 ± 0.5 years. In addition to cross-sectional studies, D'Ascenzi et al. (2017) found increased RV outflow tract and RV basal end-diastolic diameter in swimmers compared to controls. These data indicate that cardiac adaptation to exercise starts at a young age.

Exercise training strains both cardiac dimensions and vascular properties; evidence shows that athletes have alterations in vascular structures and function. In adults, the concept of an athlete's artery is discussed that postulates an association between endurance activities and reduced arterial wall thickness with a simultaneously enlarged arterial lumen, and better arterial function at all ages (Green et al., 2012, 2017). In contrast, adults performing strength sports seem to have increased arterial wall thickness and enlarged arterial lumen (Feairheller et al., 2016). Aerobic exercise seems to have a greater impact on the stiffness of peripheral (conduit) arteries such as the common carotid artery (CCA) compared to the aorta (Ashor et al., 2014). In normally active children and adolescents, improved endothelial function and arterial wall thickness have been reported (Edwards et al., 2012; Idris et al., 2015).

This study aimed to determine the arterial structure and function of young athletes 12–17 years old and compare these parameters to reference values of a healthy cohort. We hypothesized that young athletes have better vascular properties, defined by lower carotid intima-media thickness (cIMT), increased arterial elasticity, and lower arterial stiffness.

## Materials and Methods

This prospective study was conducted between November 2017 and September 2020 at our pediatric sports medical outpatient department. All participants and their legal guardians gave written informed consent to participate in this study. The study was approved by the local ethics committee (project numbers 301/18 S and 131/19 S-SR).

### Study Subjects

**Figure 1:**
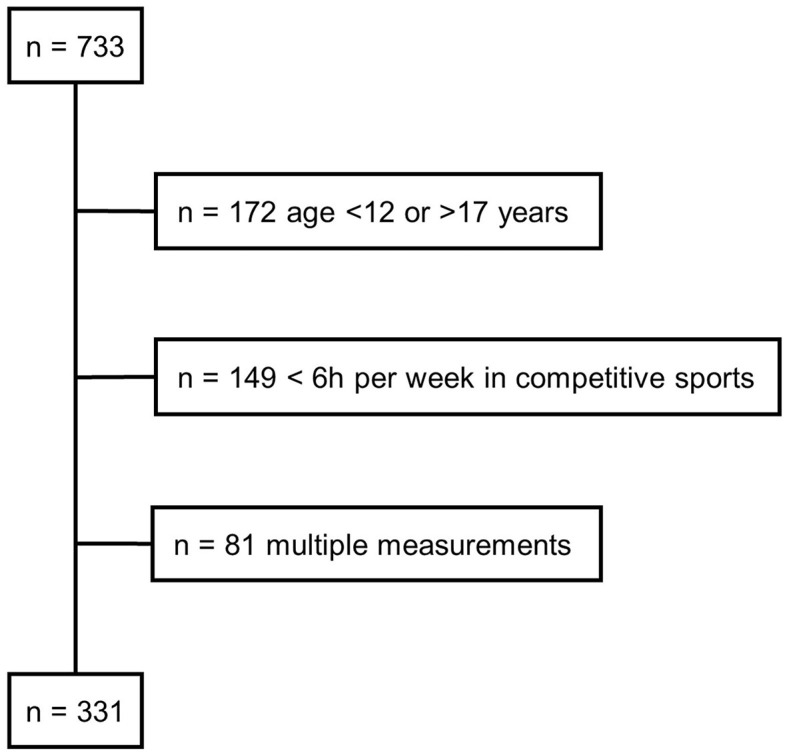
Flowchart of inclusion and exclusion of study subjects.

Children and adolescents visited our outpatient department for a pre-participation screening and ultrasound measurement of the CCA. The children and adolescents were enrolled into this study via their regular pre-participation screening. They were not recruited specifically, but voluntarily agreed to the examination in order to receive information about their sports aptitude. Therefore, the inference of our sample to the overall population of young athletes is limited. Of the 733 patients screened, 412 met the following inclusion criteria: age between 12 and 17 years, no acute infection, absence of cardiovascular or metabolic diseases, and participation in a competitive sports club activity for at least 6 h per week in addition to leisure-time physical activities and physical education. Of the 412 young athletes, 81 were measured twice, but only the first examination was included in the analysis. Thus, the final data set was composed of 331 young athletes (77 female, Figure 1). The athletes participated in the following sports club activities: soccer (*n* = 127), volleyball (*n* = 41), hockey (*n* = 33), wrestling (*n* = 20), basketball (*n* = 17), handball (*n* = 15), cross-country skiing (*n* = 13), rowing (*n* = 13), swimming (*n* = 10), judo (*n* = 6), track and field (*n* = 5), billiard (*n* = 4), ski jumping (*n* = 4), biathlon (*n* = 3), jiu-jitsu (*n* = 3), skiing (*n* = 3), cycling (*n* = 2), gymnastics (*n* = 2), snowboard (*n* = 2), climbing (*n* = 1), cycle ball (*n* = 1), ice hockey (*n* = 1), running (*n* = 1), sailing (*n* = 1), synchronized swimming (*n* = 1), tennis (*n* = 1), and triathlon (*n* = 1). The study participants were requested that the last food intake should be approximately 1 hour before the examination. In addition, no intensive training should take place 48 h before the examination. None of the young athletes reported smoking.

### Brachial Blood Pressure and Central Arterial Stiffness

Brachial systolic (bSBP), diastolic blood pressure (bDBP), and central arterial stiffness were examined with a single measurement using a noninvasive oscillometric device (Mobil-O-Graph®, IEM, Stolberg, Germany). Measurements were taken from the left upper arm in a supine position after 10 min of rest. Cuff size was determined according to the upper arm circumference (XS, 14–20 cm; S, 20–24 cm; M, 24–32 cm; L, 32–38 cm; XL, 38–55 cm). Central systolic blood pressure (cSBP) and aortic pulse wave velocity (aPWV) were calculated indirectly from pulse wave assessed at the brachial artery using the ARCSolver Algorithm (Austrian Institute of Technology, Vienna, Austria) (Wassertheurer et al., 2010). Standard deviation scores (SDS) for bSBP and bDBP were calculated according to the German reference values of Neuhauser et al. (2013). The SDS for cSBP and aPWV were calculated according to the German reference values of Elmenhorst et al. (2015).

### Carotid Structure and Function

The cIMT was measured semi-automatically using B-mode ultrasound 1 cm proximal to the bifurcation of the CCA, according to the recommendations of the Association for European Pediatric Cardiology (Dalla Pozza et al., 2015). Patients were positioned in a supine position, with a slightly extended neck and head turned 45° opposite to the side being investigated. After 10 min of rest, four end-diastolic cIMT measurements were taken on the far wall at two angles on the left (210° and 240°) and two angles on the right (120° and 150°) side. Using a three-lead electrocardiogram (ECG), the end-diastolic phase was determined.

Carotid function and diameter were determined using the echo-tracking method in M-mode ultrasound in the same position as the cIMT measurement. Tracking gates, placed at the CCA near and far wall, tracked the wall motion during five consecutive heart cycles. Carotid diameter in systole (D_max_) and diastole (D_min_) were measured and parameters for elasticity and stiffness were calculated according to the following formulas:

(1) $$\begin{array}{r} Arterial\;compliance\;\left(AC\right)\;=\;\pi \;\frac{\left(D_{\overset{2}{\max}}-\;D_{\overset{2}{\min}}\right)}{4^{*}\left(bSBP-\;bDBP\right)} \end{array}$$

(2) $$\begin{array}{r} Elastic\;modulus\;\left(Ep\right)\;=\;\frac{bSBP-\;bDBP}{\frac{D_{\max}-\;D_{\min}}{D_{\min}}} \end{array}$$

(3) $$\begin{array}{r} \beta \;stiffness\;index\;\left(\beta \right)\;=\;\frac{\ln\frac{bSBP}{bDBP}}{\frac{D_{\max}-\;D_{\min}}{D_{\min}}} \end{array}$$

(4) $$\begin{array}{r} Carotid\;pulse\;wave\;velocity\;\left(PWV\beta \right)\;=\;\sqrt{\frac{\beta^{*}bDBP}{2\rho }} \end{array}$$

Carotid diastolic diameter was used to calculate the cIMT÷carotid diameter ratio (cIDR). All parameters were calculated as the average of four measurements. SDS were calculated using German reference values, which were assessed with the same protocol and equipment (ProSound Alpha 7 with a linear array of 5–13 MHz, Hitachi Healthcare, Tokyo, Japan) (Weberruß et al., 2015; Semmler et al., 2020).

### Physical Activity and Performance

Weekly training duration and intensity were recorded using the MoMo Physical Activity Questionnaire for pupils (Schmidt et al., 2016). All athletes reported the time (min/week) they spent exercising in their sport, the number of months they performed this amount of training for the past year, and the subjective intensity (low, moderate, and high). By multiplying the time and the ratio of the number of months divided by 12, the training duration per week was determined. Depending on the intensity, each type of sport was assigned an individual MET value (Ridley et al., 2008; Ainsworth et al., 2011). To calculate the MET-hours-index, the MET values were multiplied by the training duration and divided by 60. The training experience was assessed based on the number of years the young athletes have practiced their sport on a competitive level.

Physical performance of the young athletes was measured using a standard cardiopulmonary exercise test on an electronically braked cycle ergometer (Lode Excalibur, Lode B.V., Groningen, Netherlands), following international criteria (American College of Sports Medicine, 2014; Massin, 2014). Each young athlete performed a standardized ramp protocol adapted by Godfrey (1974), after a 2-min warm-up period. Ramp inclination was determined by calculating the estimated maximum performance in W/kg (Paridon et al., 2006) and the estimated exhaustion after 10 ± 2 min (Takken et al., 2017). Subjects performed the exercise test at a pedal rate of 70–80 revolutions per minute. Maximum power output was recorded and related to body weight (W/kg). Heart rate was continuously recorded using a 12-lead ECG (CardioPart12, AMEDTEC, Aue, Germany). SDS of maximum power output were calculated using the reference values of Blanchard et al. (2018).

### Statistical Analysis

Underweight (<10th percentile), normal weight (10th−90th percentile), overweight (>90th to 95th percentile), and obesity (>95th percentile) were defined according to age- and sex-specific German reference values (Kromeyer-Hauschild, 2001). Hypertension was determined as BP >95th percentile (Neuhauser et al., 2013). Impaired arterial structure was defined as cIMT or cIDR >75th percentile (Dalla Pozza et al., 2015). Stiffness parameters >90th percentile and AC <10th percentile were defined as elevated arterial stiffness and reduced arterial elasticity, respectively (Elmenhorst et al., 2015; Urbina et al., 2019). A cSBP >90th percentile was defined as elevated cSBP (Elmenhorst et al., 2015).

Descriptive data are expressed as mean value ± standard deviation or in relative values (%). The cIMT, carotid diameter, cIDR, AC, β, Ep, PWVβ, cSBP, and aPWV are presented as raw values and SDS, according to corresponding reference values.

One investigator performed all measurements. Reproducibility of the ultrasound measurement was examined in 20 young adults measuring every individual twice. We used coefficients of variation (CV) to assess the intra-observer variability for the principal investigator and inter-observer variability between the investigator of this study and the reference collective (Weberruß et al., 2015).

After testing for normal distribution, sex differences were analyzed using unpaired Student's *t*-tests. One-sample *t*-tests were performed to investigate the deviations of SDS values compared to the value “0”. Since there were missing values for some parameters, we performed an analysis of missing values (oscillometry, ultrasound, and ergometry). Missing values were imputed using fully conditional specification with age, sex, body weight, and body height as predictors only and all variables with missing values as predictors and imputing variables. Data were analyzed using IBM SPSS version 25.0 (SPSS, Inc. Chicago, IL, USA). A *p*-value of < 0.05 was considered statistically significant.

## Results

The mean age of the 331 young athletes (23.3% female) was 14.6 ± 1.30 years. Body mass was 59.1 ± 12.4 kg and body height was 170.7 ± 11.3 cm (Table 1). The mean body height SDS was 0.37 ± 1.14 (Table 2). 16 subjects (4.8%) were underweight, 15 (4.5%) were overweight, and 2 (0.6%) were obese. Participants exercised 565.6 ± 206.0 min/week in their sport, and the training duration was higher in girls than in boys (619.2 ± 237.6 min/week vs. 546.8 ± 192.7 min/week, *p* = 0.007). The young athletes reported a MET-hours-index of 87.7 ± 33.8. The average training experience was 3.65 ± 2.51 years. Compared to females, male young athletes showed a higher absolute (280.1 ± 65.0 W vs. 232.0 ± 36.9 W, *p* < 0.001) and relative maximum power output (4.68 ± 0.49 W/kg vs. 4.16 ± 0.52 W/kg, *p* < 0.001) in the exercise test. The SDS for maximum power output was 1.85 ± 1.15 (Table 2).

**Table 1:** Study characteristics.

	**Total (*n* = 331)**	**Boys (*n* = 254)**	**Girls (*n* = 77)**	***p***
	**M ± SD**	**M ± SD**	**M ± SD**	
Age (yrs)	14.6 ± 1.30	14.7 ± 1.31	14.6 ± 1.28	0.556
Body mass (kg)	59.1 ± 12.4	60.0 ± 13.1	56.2 ± 9.12	**0.005**
Body height (cm)	170.7 ± 11.3	172.1 ± 11.8	166.1 ± 8.34	**<** **0.001**
BMI (kg/m^2^)	20.1 ± 2.48	20.0 ± 2.52	20.3 ± 2.32	0.340
BSA (m^2^)	1.67 ± 0.23	1.69 ± 0.24	1.61 ± 0.16	**0.001**
bSBP (mmHg)	115.7 ± 9.29	116.5 ± 9.51	113.1 ± 8.00	**0.004**
bDBP (mmHg)	65.0 ± 6.20	64.9 ± 6.28	65.5 ± 5.97	0.440
Training duration (min/week)	565.6 ± 206.0	546.8 ± 192.7	619.2 ± 237.6	**0.007**
Training intensity (MET-hours-index)	87.7 ± 33.8	85.8 ± 29.1	94.2 ± 45.9	0.138
Training experience (years)	3.65 ± 2.51	3.70 ± 2.52	3.50 ± 2.48	0.550
Maximum power output (W)	268.9 ± 63.0	280.1 ± 65.0	232.0 ± 36.9	**<** **0.001**
Maximum power output (W/kg)	4.56 ± 0.54	4.68 ± 0.49	4.16 ± 0.52	**<** **0.001**
cIMT (mm)	0.48 ± 0.04	0.49 ± 0.04	0.47 ± 0.04	**<** **0.001**
Carotid diameter (mm)	5.65 ± 0.45	5.68 ± 0.46	5.56 ± 0.40	**0.034**
cIDR (%)	0.09 ± 0.01	0.09 ± 0.01	0.08 ± 0.01	0.174
AC (mm^2^/kPa)	1.52 ± 0.33	1.53 ± 0.33	1.49 ± 0.35	0.294
β	3.23 ± 0.72	3.23 ± 0.71	3.23 ± 0.73	0.934
Ep (kPa)	37.9 ± 8.70	37.9 ± 8.55	37.8 ± 9.26	0.956
PWVβ (m/s)	3.61 ± 0.37	3.61 ± 0.36	3.63 ± 0.42	0.652
cSPB (mmHg)	106.5 ± 10.1	107.4 ± 10.4	103.3 ± 7.92	**<** **0.001**
aPWV (m/s)	4.96 ± 0.43	5.02 ± 0.44	4.79 ± 0.32	**<** **0.001**

*BMI, body mass index; BSA, body surface area; bSBP, brachial systolic blood pressure; bDBP, brachial diastolic blood pressure; MET, metabolic equivalent; cIMT, carotid intima-media thickness; cIDR, carotid intima-media thickness÷carotid diameter ratio; AC, arterial compliance; β, beta stiffness index; Ep, elastic modulus; PWVβ, carotid pulse wave velocity; cSBP, central systolic blood pressure; aPWV, aortic pulse wave velocity. Significant results are marked in bold*.

**Table 2:** SDS values of parameters of body height, BMI, blood pressure, physical performance and vascular properties.

	**M ± SD**	***p* value**
Body height SDS	0.37 ± 1.14	**<** **0.001**
BMI SDS	0.02 ± 0.77	0.684
bSBP SDS	0.03 ± 0.95	0.561
bDBP SDS	−0.45 ± 0.91	**<** **0.001**
Maximum power output SDS	1.85 ± 1.15	**<** **0.001**
cIMT SDS	0.37 ± 1.34	**<** **0.001**
Carotid diameter SDS	0.08 ± 0.98	0.133
cIDR SDS	0.20 ± 1.41	**0.009**
AC SDS	0.99 ± 0.83	**<** **0.001**
β SDS	−0.75 ± 0.88	**<** **0.001**
Ep SDS	−0.88 ± 0.93	**<** **0.001**
PWVβ SDS	−1.04 ± 0.97	**<** **0.001**
cSBP SDS	0.42 ± 1.29	**<** **0.001**
aPWV SDS	0.55 ± 1.33	**<** **0.001**

*BMI, body mass index; bSBP, brachial systolic blood pressure; bDBP, brachial diastolic blood pressure; cIMT, carotid intima-media thickness; cIDR, carotid intima-media thickness÷carotid diameter ratio; AC, arterial compliance; β, beta stiffness index; Ep, elastic modulus; PWVβ, carotid pulse wave velocity; cSBP, central systolic blood pressure; aPWV, aortic pulse wave velocity. Significant results are marked in bold*.

### Intra- and Inter-Observer Variability

Table 3 shows the intra- and inter-observer variability of ultrasound measurements. Intra-observer variability was lower than inter-observer variability. Both intra- and inter-observer CV were lowest in cIMT (0.58 and 2.09). PWVβ had the lowest intra- and inter-observer CV of all stiffness parameters (1.46 and 5.57). Intra-observer CV was highest in Ep (4.49) and inter-observer CV was highest in β (13.54).

**Table 3:** Intra- and inter-observer reliability of ultrasound parameters (coefficient of variation).

	**Intra-observer**	**Inter-observer**
cIMT	0.58	2.09
Carotid diameter	0.87	3.63
cIDR	0.81	4.38
AC	1.63	6.59
β	3.40	13.54
Ep	4.49	13.11
PWVβ	1.46	5.57

*cIMT, carotid intima-media thickness; cIDR, carotid intima-media thickness÷carotid diameter ratio; AC, arterial compliance; β, beta stiffness index; Ep, elastic modulus; PWVβ, carotid pulse wave velocity*.

### Parameters of Arterial Structure

Study participants had a mean cIMT of 0.48 ± 0.04 mm, a carotid diameter of 5.65 ± 0.45 mm, and a cIDR of 0.09 ± 0.01% (Table 1). Boys had thicker cIMT (0.49 ± 0.04 mm vs. 0.47 ± 0.04 mm, *p* < 0.001) and larger carotid diameters (5.68 ± 0.46 mm vs. 5.56 ± 0.40 mm, *p* = 0.034) than girls. The cIDR was similar between sexes. A total of 135 young athletes (40.8%) had cIMT >75th percentile, 88 (26.6%) had carotid diameter >75th percentile, and 124 (37.5%) exhibited cIDR >75th percentile.

### Parameters of Arterial Function

The bSBP was significantly higher in boys than in girls (116.5 ± 9.51 mmHg vs. 113.1 ± 8.00 mmHg, *p* = 0.004, Table 1). Systolic and diastolic hypertension were observed in 17 (5.1%) and 3 (0.9%) subjects, respectively. Boys had higher cSBP (107.4 ± 10.4 mmHg vs. 103.3 ± 7.92 mmHg, *p* < 0.001) and aPWV (5.02 ± 0.44 m/s vs. 4.79 ± 0.32 m/s, *p* < 0.001) than girls. AC, Ep, β, and PWVβ were not significantly different. A total of 71 young athletes (21.5%) had cSBP >90th percentile and 94 (28.4%) had aPWV >90th percentile.

### Comparison of Structural and Functional Parameters to Normative Values

**Figure 2:**
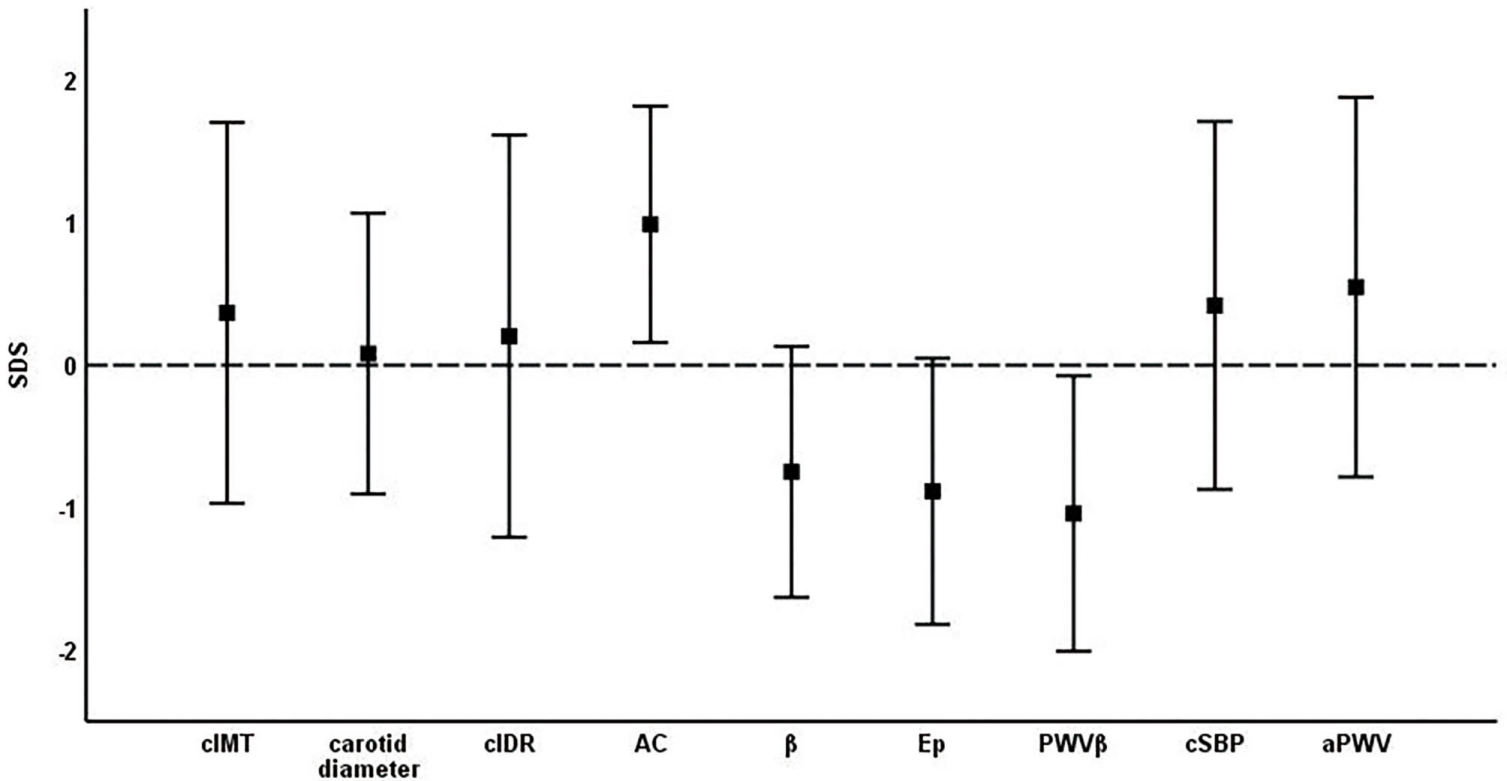
Mean ± standard deviation of SDS of vascular parameters. cIMT, carotid intima-media thickness; cIDR, carotid intima-media thickness÷carotid diameter ratio; AC, arterial compliance; β, beta stiffness index; Ep, elastic modulus; PWVβ, carotid pulse wave velocity; cSBP, central systolic blood pressure; aPWV, aortic pulse wave velocity.

Young athletes showed thicker cIMT SDS (0.37 ± 1.34, *p* < 0.001) and higher cIDR SDS (0.20 ± 1.41, *p* = 0.009) (Table 2, Figure 2). Study participants had improved arterial elasticity, as indicated by the AC SDS (0.99 ± 0.83, *p* < 0.001) and reduced peripheral arterial stiffness parameters, including β SDS (−0.75 ± 0.88, *p* < 0.001), Ep SDS (−0.88 ± 0.93, *p* < 0.001), and PWVβ SDS (−1.04 ± 0.97, *p* < 0.001) compared to the reference cohort. In contrast, central stiffness parameters cSBP SDS (0.42 ± 1.29, *p* < 0.001) and aPWV SDS (0.55 ± 1.33, *p* < 0.001) were higher in the young athletes compared to reference values.

In a sub analysis with strength endurance athletes only (*n* = 241), cIMT SDS (0.46 ± 1.36, *p* < 0.001), cIDR SDS (0.35 ± 1.37, *p* < 0.001), AC SDS (1.06 ± 0.83, *p* < 0.001), β SDS (−0.84 ± 0.87, *p* < 0.001), Ep SDS (– 1.00 ± 0.93, *p* < 0.001), PWVβ SDS (−1.16 ± 0.96, *p* < 0.001), cSBP SDS (0.40 ± 1.21, *p* < 0.001), and aPWV SDS (0.55 ± 1.27, *p* < 0.001), but not carotid diameter (−0.01 ± 0.98, *p* = 0.990) were significantly different compared to “0”.

## Discussion

To the best of our knowledge, this is the first study to investigate the vascular structure and function in young athletes. The cIMT and cIDR were higher in young athletes compared to reference cohorts. Furthermore, young athletes presented better carotid elasticity and lower carotid stiffness (β, Ep, PWVβ) but higher central arterial stiffness (cSBP, aPWV) compared to their peers.

The young athletes were physically active for 565.6 ± 206.0 min/week in sports clubs, which was about 6 h longer than individuals in a representative German sample (Rauner et al., 2015). The maximum power output with an SDS of 1.85 ± 1.15 was higher than the values in the reference population of Blanchard et al. (2018). Therefore, we assume that the young athletes in this study are more physically active and efficient than average.

In our study, we found higher cIMT and cIDR compared to a healthy reference cohort and 40.8% of the young athletes had cIMT >75th percentile. The results of our study contradict Thijssen et al. (2012), who concluded that exercise has no significant effect on vascular properties in the young. Adaptation to exercise is well described in adult athletes; however, only one study investigated cIMT in adolescent athletes. This study demonstrated that cIMT was lower in adolescent wrestlers than in controls (Demirel et al., 2019). However, the large cIMT deviation in the study by Demirel et al. (2019) relative to our study may have impaired the comparability of the two studies; additionally, different ultrasound devices were used in the two studies which also limits comparability. The reference values of Weberruß et al. (2015) are age- and sex-specific. However, cIMT also increases with higher body height (Doyon et al., 2013). Since our study population has a significantly higher body height SDS (see Table 2), it cannot be excluded that the higher body size influences the high prevalence of cIMT >75th percentile.

According to Liu et al. (2015), maximum, mean, and minimum arterial diameters in young adult basketball players were improved compared to sedentary controls. We did not observe significant changes in arterial diameter in our population of young athletes compared to the reference cohort. Remodeling of the arterial diameter may be dependent on the physically active limb (Green et al., 2017). Huonker et al. (2003) showed that the diastolic diameter of the subclavian artery was higher in tennis players and that the diastolic diameter of the femoral artery was higher in cyclists than in untrained controls. Furthermore, in below-knee amputated athletes, the diastolic diameter of the femoral artery in the intact limb was higher than on the lesion side (Huonker et al., 2003). Approximately two-thirds of the young athletes in our study exercised in sports involving lower limb movement. To investigate local remodeling due to exercise in young people, the examined artery should be selected based on the type of exercise performed.

Carotid function improved in our athletic population compared to a healthy reference cohort, indicated by higher carotid elasticity (AC) and reduced carotid stiffness (β, Ep, and PWVβ). Our results are in agreement with Nishiwaki et al. (2017), who reported lower cardio-ankle vascular index in young adult cyclists and reduced brachial-ankle PWV in both cyclists and swimmers than in controls. Furthermore, ultrasound-derived β and Ep of the CCA were lower in ten young adult basketball players than in sedentary controls (Liu et al., 2015). In addition, higher flow-mediated dilation was observed in young adult athletes than in inactive controls (Podgorska et al., 2017).

However, central hemodynamic parameters, including aPWV and cSBP, were higher in our cohort compared to the appropriate reference values, indicating higher aortic stiffness in young athletes. The same vascular effects were observed in middle-aged marathon runners. Participants in this study had significantly higher bSBP, bDBP, aortic SBP, aortic DBP, and PWV compared to controls (Vlachopoulos et al., 2010). Coates et al. (2018) compared vascular changes after 3 weeks of regular exercise training (regular training load) and 3 weeks of overload training (150% of regular training load) in 26 endurance athletes between 18 and 50 years; carotid-femoral PWV significantly increased in the overload group, indicating that overload training induced central arterial stiffening. Because aPWV and BP are not only age- and sex- but also height-dependent, it cannot be excluded that the higher body height in our study population influences the higher SDS values of aPWV and cSBP (Reusz et al., 2010; Regnault et al., 2014).

Sport types, especially endurance and strength training, have different impacts on vascular function. While endurance exercise is associated with a decrease in arterial stiffness, athletes participating in strength exercise exhibit elevated arterial stiffness (Bertovic et al., 1999; Otsuki et al., 2007; D'Andrea et al., 2012). During the competitive season, bSBP and bDBP increased in American-style football players and central aortic pulse pressure and PWV were significantly higher after season, measured by applanation tonometry (Kim et al., 2015). Furthermore, powerlifting athletes showed reduced cSBP after a 12-week strength program (Jürgenson et al., 2019). In contrast to our findings, Demirel et al. (2019) compared ultrasound measurements of the CCA in adolescent wrestlers and sedentary controls and found no significant differences in distensibility, elastic modulus, and compliance of the CCA. The differences in the study population (young athletes of various types of sport vs. adolescent wrestlers) between our study and Demirel et al. (2019) could explain the discrepancies in these results.

In addition to the distinction between endurance and strength sports, training experience may also influence arterial stiffness. Otsuki et al. (2007) examined endurance-trained men with a short and a long career in competitive sports vs. strength-trained men with a short and long career in competitive sports and compared results to sedentary controls. Endurance athletes with a long competitive career, but not those with a short competitive career, had lower aPWV than sedentary controls. Furthermore, aPWV was higher in strength athletes with both short and long competitive careers than in sedentary controls. The authors concluded that longer experience in endurance training positively influenced aPWV, but the higher the number of strength exercises within a sporting activity, the higher (worse) the aPWV.

The classification of different types of sports into “endurance sports” or “strength sports” categories is difficult. For example, ball sports, like soccer or basketball, predominantly consist of an endurance component but also involve strength components. Athletic training has gained importance, even at a young age, as a means of improving cardiopulmonary and muscular endurance and high-speed strength. The young athletes in our study mainly participated in cardiopulmonary endurance sports and had trained for 3.65 ± 2.51 years. This might explain the higher aPWV and cSBP in our study population. A comparison of young athletes participating in endurance, muscular endurance, or strength sports could provide detailed information on possible differences in arterial stiffness between sport types.

40.8% of young athletes showed cIMT >75th percentile. The high cIMT could indicate preliminary stages of atherosclerosis in young athletes and, thus, a negative effect of competitive sports on these vessels. The development of atherosclerosis begins in childhood and adolescence, when low-density lipoprotein particles enter and accumulate in the intimal layer. During the inflammatory process, endothelial cells are activated and secrete adhesion molecules, leading to the formation of foam cells via a complex process (Insull, 2009). Besides morphometric alterations of the arterial wall, including intimal thickening, the atherosclerotic process also leads to a decreased endothelial function and, therefore, increase in arterial stiffness. Therefore, arterial wall thickening and reduced elasticity indicate that competitive sports in adolescence may have a pathological effect on vascular health. However, young athletes exhibited improved elasticity and wall thickening and higher cIDR. Arterial segments with higher wall-to-lumen ratios have better arterial function (Thijssen et al., 2011). The underlying mechanism seems to be the greater number of VSMC in the arterial wall and an enhanced response to vasodilating stimuli (Thijssen et al., 2011; Green et al., 2017). Thus, the thickening of the intima-media complex in combination with improved carotid function indicates VSMC proliferation in the tunica media, improved vascular reactivity and, therefore, a functional adaptation to exercise.

### Limitations

This study has several limitations. First, aortic stiffness was only estimated via the ARCSolver Algorithm and not directly measured. A recent report on the technical effectiveness of the ARCSolver Algorithm showed that cSBP measurement is reliable, but aPWV is slightly overestimated (EUnetHTA OTCA-24 Assessment Team, 2020). Second, the methodology of measuring bSBP and bDBP in our study (Mobil-O-Graph) differs from the measurement in the reference population (Datascope Accutorr Plus). Sarganas et al. (2020) compared the BP values of both devices and found that the Mobil-O-Graph provided higher values than the Datascope Accutorr Plus. Although the validation was only carried out in adults aged 21 and older, it is possible that this tendency is already evident in children and adolescents. Third, pubertal status was not assessed and, therefore, not included in the analysis as a factor influencing the results. Although it is known that pubertal status influences vascular properties (Drole Torkar et al., 2020), it was not possible to include the assessment via Tanner Scale into this setting due to child protection concerns (McClean et al., 2018). Fourth, the types of sports are unequally distributed within our study population. This limitation is related to patient recruitment. Even though the associations of different types of sports support and suggest pre-participation screening, mainly male soccer players were included in the study. Young athletes from other types of sports and girls are underrepresented. Fifth, CCA, as a parameter of arterial structure and function, is used for generalized systemic adaptations to exercise. Different types of sports can have a different impact on CCA, but they could have also stimulated local arteries (e.g., brachial or femoral). Sixth, we did not consider the proportion of exercise training spent in endurance vs. strength sports and the timing of the examination (preseason, during season, and postseason), although both aspects are known to influence cardiac adaptation or the adaptation of physical performance to exercise (Dores et al., 2015; Emmonds et al., 2020). Therefore, we can conclude in general that young athletes differ in their vascular properties from controls, but we are not able to conclude that strength and/or endurance athletes differ from the reference collective. In order to investigate the long-term effects of different types of sports and training levels on vascular parameters in young athletes, we are currently conducting a long-term study (Baumgartner et al., 2019).

With regard to intra-observer variability, the scatter between the two measurements (CV <5% in all parameters) was very low; the intra-observer CV of the structural parameters was slightly lower than that of the functional parameters. Compared to previous studies, the intra-observer variability of structural and functional parameters was lower (Kanters et al., 1998; Iannuzzi et al., 2008; Böhm et al., 2009; Bjällmark et al., 2010). The inter-observer variability of the structural parameters was also lower in our study compared to the literature (Böhm et al., 2009). The CV of the inter-observer variability of stiffness parameters β and Ep was higher compared to Weberruß et al. (2015) but lower than Kanters et al. (1998). In summary, both intra- and inter-observer variabilities of the ultrasound parameters were acceptable to confirm the good quality of the data.

## Conclusion

In summary, we observed improved carotid elasticity but altered central hemodynamics and carotid structure in young athletes compared to a reference cohort. The investigation of vascular properties in young athletes is new and rarely performed. The paucity of studies in young athletes is due to the perceived lack of medical need for these measurements in young athletes, in contrast to ECG or echocardiography. However, our results indicate that competitive sports in youth can alter vascular properties.

## Data Availability Statement

The datasets analyzed within the study are available from the corresponding author on reasonable request.

## Ethics Statement

The studies involving human participants were reviewed and approved by the Technical University of Munich (project numbers 301/18 S and 131/19 S-SR). Written informed consent to participate in this study was provided by the participants' legal guardian.

## Author Contributions

LB, HW, TS, and RO-F designed the study. LB, KA, TE, and DG performed the data collection. LB performed the data analysis and drafted the manuscript. All authors reviewed, edited, and approved the manuscript.

## Conflict of Interest

The authors declare that the research was conducted in the absence of any commercial or financial relationships that could be construed as a potential conflict of interest.
